# Treatment of High-Risk Neuroblastoma

**DOI:** 10.3390/children10081302

**Published:** 2023-07-28

**Authors:** Julie Krystal, Jennifer H. Foster

**Affiliations:** 1 Zucker Hofstra School of Medicine, Department of Pediatrics, Cohen Children’s Medical Center, New Hyde Park, NY 11040, USA; 2 Department of Pediatrics, Baylor College of Medicine, Texas Children’s Cancer Center, Houston, TX 77030, USA; jhfoster@texaschildrens.org

**Keywords:** neuroblastoma, pediatric oncology, solid tumor

## Abstract

High-risk neuroblastoma is a highly aggressive solid tumor that most commonly presents in early childhood. Advances in treatment through decades of clinical trials and research have led to improved outcomes. This review provides an overview of the current state of treatment for high-risk neuroblastoma.

## 1. Introduction

Neuroblastoma is the most frequent cancer in children less than one year old, and the most common solid tumor occurring outside of the central nervous system in all age groups [1]. Neuroblastoma displays a broad spectrum of clinical behavior and includes the following three risk groups: low, medium, and high. About half of patients with neuroblastoma are classified as having high-risk disease, by a combination of factors including age at diagnosis, extent of disease, histological findings, and cytogenetic characteristics such as MYCN amplification and DNA ploidy. Historically, this group of patients had dismal outcomes, but with current multi-modal therapy, about half of children can be expected to survive [2]. High-risk neuroblastoma (HRNBL) treatment is actively evolving and currently includes intensive chemotherapy, radiation therapy, autologous stem cell transplant (ASCT), and immunotherapy. Research efforts are currently focused on identifying more targeted treatments, reducing toxicity and late effects, and improving outcomes for these challenging patients through novel approaches.

## 2. Defining High-Risk Neuroblastoma

The International Neuroblastoma Risk Group (INRG) developed a staging system in 2005 that was unrelated to initial surgical resection and different from its predecessor, the International Neuroblastoma Staging System (INSS) [3,4]. The International Neuroblastoma Risk Group staging system (INRGSS) uses a set of imaging-defined risk factors (IDRFs) to separate non-metastatic tumors as L1 (no IDRFs present) or L2 (with IDRFs). Patients with metastatic disease are classified as stage M, with the exception of children younger than 18 months with metastatic disease limited to skin, liver, and/or bone marrow (stage MS). The INRG stage, in addition to the patients age at diagnosis, histology and cytogenetic features are combined to determine the patients overall risk group. The Children’s Oncology Group (COG) released an updated risk classifier in 2021 based on the INRGSS and incorporated segmental chromosomal aberrations (SCA) as an additional biomarker affecting risk group [5]. The SCAs that are the most well-studied in neuroblastoma include chromosome deletions of 1p and 11q, and gains in chromosomes 1q, 2p, and 17q. The presence of SCAs is associated with worse outcomes, as well as with age >18 months and advanced stage disease [6]. MYCN, an oncogene that is found to be amplified in around 25% of neuroblastomas, correlates with a poor prognosis and indicates a high-risk designation in all scenarios [7]. Mutations of the anaplastic lymphoma kinase gene (ALK) occur in around 5–10% of neuroblastoma cases and are associated with poor overall survival [8,9]. Metastatic disease in children older than 18 months is considered high-risk regardless of their other features, as this group has historically done poorly. While children with low-risk and intermediate-risk neuroblastoma have excellent outcomes with 5-year overall survival over 95%, while 5-year year overall survival in those with high-risk disease remains about 60% [5]. Appropriate risk classification remains an important aspect of approaching neuroblastoma to best define the subset of patients who require intensive multi-modal therapy.

## 3. Frontline Treatment

**Figure 1:**
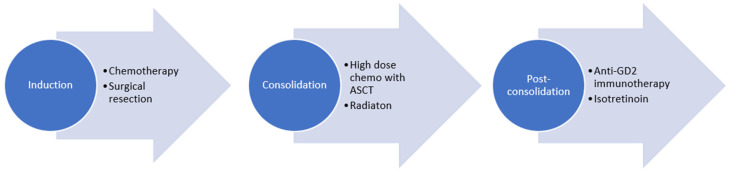
Treatment of high-risk neuroblastoma.

Most North American institutions and international pediatric oncology collaborative groups follow a treatment plan with induction chemotherapy, during which the primary tumor is surgical resected. This is followed by consolidation with ASCT and radiotherapy, and post-consolidation treatment with anti-GD2-based immunotherapy and isotretinoin (Figure 1). The total upfront treatment duration is approximately 18 months.

### 3.1. Induction

The goal of induction is to eliminate visible disease and achieve remission. Induction regimens typically include five to eight cycles of intensive chemotherapy including alkylators, platinum, and topoisomerase drugs. Current COG trials utilize five cycles with topotecan, vincristine, doxorubicin, cyclophosphamide, cisplatin, and etoposide (NCT03126916). The Society of Pediatric Oncology Europe Neuroblastoma Group (SIOPEN) continues to utilize a rapid COJEC regimen that gives eight cycles of compressed chemotherapy with combinations of vincristine, carboplatin, etoposide, cyclophosphamide, and cisplatin. Trials comparing rapid COJEC to non-compressed induction therapy have shown similar outcomes, and this remains the standard induction regimen in Europe [10].

In order to prepare for ASCT, patients undergo stem cell collection during induction. Metastatic disease in the bone marrow is present at diagnosis in many patients with HRNBL, and they may have residual marrow disease at the time of stem cell collection. These collected stem cells can undergo a process of purging to remove any remaining neuroblastoma cells, so that these malignant cells will not be returned to the patient. A COG study examined the use of autologous stem cells that had been purged after collection compared to the use of unpurged cells and found no different in survival between the groups [11]. It is therefore standard practice to use unpurged cells. During collection it is important to collect enough cells for both ASCT as well as potential future therapies requiring stem cells, such as I-^131^ MIBG therapy.

Surgery is an important part of up-front therapy, and surgical excision of the primary tumor typically occurs during induction after several cycles of chemotherapy. This approach allows for optimal tumor shrinkage thus potentially minimizing surgical morbidity. The extent of tumor removal is important, as patients with either a complete or gross total resection have better outcomes than patients with lesser resections. Data from COG study A3973 demonstrated an improved 5-year EFS (45.9% compared to 37.9%) and lower cumulative incidence of local progression (8.5% compared to 19.8%) when there was >90% tumor resection compared to less than 90% resection [12]. A large meta-analysis examining over 1900 subjects found that there were no statistically significant differences in 5-year EFS or OS among those who had a gross total resection compared to those who had a complete tumor resection (*p* = 1.0). There was also a trend towards more intra- and post-operative complications for those who underwent gross total resection or complete tumor resection compared to those who had subtotal resections or biopsy only, as aggressive surgical resection can often be anatomically challenging and threaten vital organs or structures [13]. An analysis of over 1500 children treated on the SIOPEN HR-NBL1 study found that patients who had complete macroscopic excision had improved 5-year EFS (40% vs. 33%) and OS (45% vs. 37%) compared to those who had incomplete macroscopic resection. CLIP was also lower after complete macroscopic excision in comparison to incomplete macroscopic excision (17% vs. 30%). These differences in outcome persisted with the addition of immunotherapy [14]. Because of the aggressive nature of HRNBL, there is a difficult balance between attempting complete removal of all of the tumor and leaving residual tumor to avoid potential complications. IDRFs have been found to be useful predictors of surgical risk, indicating more challenging resections when present [15]. In assessing the effect of tumor resection on outcomes, there is likely a complex interplay between many factors including the tumors’ biological behavior and response to therapy, which makes it challenging to examine the role of surgery in isolation.

Despite the intensity of induction treatment, by the end of induction around 10% of patients will have progressive disease and only around 20% will have a complete response. Several studies have demonstrated that end-of-induction response is predictive of outcome, with the updated 2017 International Neuroblastoma Response Criteria (INRC) being validated to demonstrate this as well [16]. The INRC (Table 1) incorporates assessment of the primary tumor, metastatic disease in bone and soft tissue, and the bone marrow to provide an overall response assessment that allows for the uniform assessment of patients across treatments and studies [17].

Patients who have a poor response to induction therapy have a dismal outcome. A variety of treatments have been tried to improve outcomes within this cohort of patients. A retrospective study examining post-induction therapy for 201 patients who achieved a partial response (PR) or worse at the end of induction found that, in a cohort of patients who went on to receive bridge therapy that included dinutuximab, temozolomide, and irinotecan and/or ^131^I- MIBG prior to receiving ASCT, EFS was significantly improved among patients with stable disease (SD) in metastatic sites [18].

Efforts are underway to improve induction response (Table 2), including the currently open COG trial ANBL1531, which randomizes patients with MIBG-avid, ALK wild-type disease to standard 5-cycle induction chemotherapy or standard induction chemotherapy with the addition of high-dose ^131^I-MIBG therapy (NCT03126916). ANBL1531 is also studying the addition of targeted therapy for those children with an ALK-mutation at diagnosis, given in addition to standard therapy. A single-center trial evaluating an intensified 4-cycle induction is ongoing (NCT04947501). Studies have also examined incorporating immunotherapy into induction. ANBL17P1, a prospective single-arm pilot study, administered dinutuximab with GM-CSF during induction cycles three through five for newly diagnosed patients (NCT03786783). Forty-two patients were treated, and the combination was found to be tolerable and feasible. The overall end-of-induction response rate (including complete response, partial response, and minor response) was 86.8% [19]. A study at St. Jude Children’s Research Hospital (NCT01857934) similarly added anti-GD2 antibody with GM-CSF and interleukin-2 to six cycles of induction therapy. Among the 63 evaluable patients, the treatment was feasible and 97% had an end-of-induction partial response or better, with no patients experiencing progressive disease during induction. Patients went on to receive consolidation with ASCT and radiation therapy, and post-consolidation immunotherapy with anti-GD2 antibody and GM-CSF, and had a 3-year EFS of 73.7% and OS of 86% [20]. These encouraging results have led to the development of a large phase 3 trial of immunotherapy during induction, which is upcoming from the Children’s Oncology Group.

### 3.2. Consolidation

The goal of the consolidation phase is to eliminate any remaining minimal residual disease or remaining gross disease and consists of ASCT and radiation therapy. During ASCT, high doses of chemotherapy are given causing severe myelosuppression, after which the patient is given their stored stem cells to allow for bone marrow recovery. After data showed improved outcomes for patients who received ASCT during consolidation compared to continuation chemotherapy [21,22], a randomized phase 3 trial to compare single ASCT (CEM) to tandem ASCT (TC/CEM) during consolidation was conducted by the COG. Patients who were assigned to receive tandem ASCT had improved 3-year EFS compared to those who received single ASCT (61.6% vs. 48.4%). For patients who went on to receive immunotherapy in the post-consolidation setting, those who received tandem ASCT had improved EFS and OS compared to those who received single ASCT [23]. SIOPEN continues to employ ASCT as well, though using busulfan/melphalan (Bu/Mel) conditioning regiments while COG has traditionally utilized carboplatin, etoposide, and melphalan (CEM). In the SIOPEN HR-NBL1 trial, patients were randomly assigned to receive either Bu/Mel or CEM conditioning after receiving rapid COJEC induction. Patients who were randomized to receive Bu/Mel had improved 3-year EFS compared to those who were assigned to receive CEM (50% vs. 38%) [24]. The ongoing SIOPEN HR-NBL2 trial randomizes patients to Bu/Mel single ASCT compared to tandem ASCT (NCT04221035). At this time, ASCT remains the standard approach in North America and through international cooperative groups, though given the toxicity of high-dose chemotherapy and significant late effects for survivors, future studies will need to identify novel biomarkers to determine which patients might be able to forgo ASCT, and the ability of newer targeted therapies to be used in its place.

Following ASCT, 21.6 Gy of external beam radiation is given to the preoperative primary tumor volume, as well as any sites of persistent metastatic disease present at the end of induction [25]. Attempts at intensification of radiation have not demonstrated improved outcomes. In the COG A3973 trial, patients who received broad regional nodal irradiation had no significant differences in outcomes compared to those who did not [26]. Among 323 patients who received radiotherapy in the COG ANBL0532 trial, there were 133 who had incomplete resections and were assigned to receive a boost of 14.4 Gy to gross residual primary tumor present at the end of induction. Compared to a similar historical group of patients with gross residual tumor treated on A3973 who did not receive boost radiotherapy, those who received the boost in ANBL0532 had similar outcomes [27]. Based on these results, boost radiation is no longer part of standard treatment in North America. The ongoing SIOPEN HR-NBL2 trial randomizes patients with gross residual disease to receive standard 21.6 Gy or standard radiation plus a 14.4 Gy boost to remaining tumor (NCT04221035).

While neuroblastoma is a radiosensitive disease, and radiation is critical to local control, awareness of long-term toxicity of radiation has led to studies examining reductions in radiation. A prospective trial of 25 patients who received 18 Gy to their primary tumor site did not show reduced local control or overall survival [28]. An ongoing single-arm prospective study is exploring the use of 15 Gy administered in 10 fractions given twice daily for patients who have a complete tumor resection and no remaining disease after induction (NCT02245997).

### 3.3. Post-Consolidation

Ganglioside 2 (GD2) is a glycolipid found in high levels on the outer surface of neuroblastoma cells, as well as other embryonal cancers and was identified as a targetable treatment strategy [29] The landmark phase 3 randomized COG trial ANBL0032 showed significantly improved survival in patients treated with the anti-ganglioside 2 (GD2) antibody dinutuximab in conjunction with GM-CSF and IL2, along with isotretinoin compared to isotretinoin alosne, with a 2-year EFS of 66% vs. 46% and OS of 86% vs. 75% [30]. Continued follow-up of these subjects demonstrated sustained improved outcomes with 5-year EFS and OS of 56.6% and 73.2% for those randomized to dinutuximab and 46.1% and 56.6% for those randomized to isotretinoin alone [31]. In addition to being expressed on neuroblastoma cells, GD2 is also found on peripheral nerves, resulting in high levels of neuropathic pain during treatment with anti-GD2 targeting drugs. Additional toxicities include fever, capillary leak, and allergic reactions. Most patients require an inpatient setting, and all patients require prophylactic pain medications and careful monitoring. Subsequently, the SIOPEN HR-NBL1 trial randomized patients to receive dinutuximab beta with or without IL-2 and found that patients who were assigned to not receive IL-2 had similar EFS but less toxicity than those who were assigned to receive IL-2 [32]. As a result, in both Europe and North American, anti-GD2 treatment is now given without IL-2. Following the development of dinutuximab, naxitamab, a humanized anti-GD2 antibody, was developed and studied in relapsed and refractory disease [33], leading to FDA approval for the relapsed or refractory setting.

Efforts are ongoing to find additional ways to target GD2. A phase 1 trial (NCT00911560) in 15 patients with HRNBL in second or greater remission explored a GD2/GD3-targeting vaccine and found the vaccine to be safe and tolerable. Twelve of the patients produced antibodies to GD2/GD3 and relapse-free survival was 80% at 24 months of follow-up [34]. A larger phase 2 portion of the study is now underway including patients in first complete remission.

## 4. Relapsed Disease

Outcomes remain poor in patients with HRNBL who have progressive disease or who after front-line therapy do not respond, with 4-year PFS and OS of 6% and 20%, respectively [35]. The main initial treatment for these patients include ^131^I MIBG therapy and chemoimmunotherapy with irinotecan, temozolomide, and dinutuximab. Radiopharmaceutical treatment with ^131^I-MIBG has demonstrated activity in relapsed disease either alone or in combination [36,37]. Recent studies have also shown improved response rates with a combination of dinutuximab, GMCSF, temozolomide, and irinotecan (chemoimmunotherapy). In the COG study ANBL1221 (NCT01767194), 53 patients with relapsed or refractory disease received chemoimmunotherapy with an objective response rate of 41.5%. One-year PFS and OS were 67.9% and 84.9%, respectively [38,39]. A retrospective review of 146 patients with relapsed or progressive disease who received chemoimmunotherapy demonstrated a 49% objective response rate, with 29% of those achieving a complete remission. The one-year PFS was 50%, and responses were maintained for a median of 15.9 months [40]. Based on this compelling data, chemoimmunotherapy is now being explored in the front-line setting in an ongoing COG pilot trial, ANBL19P1, where patients will receive dinutuximab, GMCSF, temozolomide, irinotecan, and isotretinoin during post-consolidation (NCT04385277).

Other strategies being explored in the relapsed setting include anti-GD2-directed chimeric antigen receptor-modified T-cells. This strategy involves collecting the patients T-cells or natural killer T-cells and modifying them ex vivo to produce a receptor targeting GD-2. These T-cells are then returned to the patient, where they may be able to induce neuroblastoma cell death. Several early phase trials using this technique are in progress (NCT02765243, NCT04539366, NCT03635632), with some preliminary data showing anti-tumor effect [41,42].

Inhibition of cyclin-dependent kinases 4/6 (CDK4/6) has been shown in preclinical work to slow neuroblastoma cell growth [43] and several trials are now exploring this strategy for patients with relapsed disease, including in combination with dintuximab, temodar, irinotecan, and GMCSF (NCT04238819, NCT05429502, NCT03709680).

Given the poor outcomes in the relapsed and refractory setting, referral for early phase trials is often appropriate when it is consistent with the patients and families goals and preferences. 

## 5. Late Effects

Children who receive HRNBL therapy are at high risk for late effects, which has significant effects on quality of life and leads to increased morbidity and mortality. Studies of survivors have found that as many as 95% have late effects including hearing loss, endocrine abnormalities, and growth issues, as well as other organ toxicity including cardiac and pulmonary [44]. Endocrinopathies, including primary hypothyroidism, growth failure and hypogonadism, may be experienced by the majority of survivors and result in the need for life-long hormone supplementation and management [45]. Survivors are also at increased risk of both hematological and solid tumor secondary malignant neoplasms [46].

One of the most frequent and deleterious late effects in neuroblastoma survivors is hearing loss, which is typically related to platinum chemotherapy. COG conducted a large study evaluating hearing loss assessments which included 53 children with neuroblastoma, and found that about half of children treated with cisplatin had ototoxicity, with severe ototoxicity ranging from 7% to 22% [47]. Because of the frequency of patients developing ototoxicity as well as its impact on learning and psychosocial outcomes, hearing loss prevention is a high priority in this patient population. A large phase 3 randomized clinical trial assigned children found to have normal hearing who had newly diagnosed cancer to receive sodium thiosulfate (STS) or observation with their planned cisplatin-containing treatment and found a significant reduction in the incidence of hearing loss in the STS group compared to the control group (28.6% vs. 56.4%, *p* = 0.00022). Cisplatin-induced hearing loss is thought to occur by the accumulation of cisplatin in the inner ear, resulting in permanent damage to the cochlea [48]. Sodium Thiosulfate can neutralize cisplatin, which is likely the mechanism for otoprotection, and thus, it is given 6 h following cisplatin infusion so that the chemotherapy is not rendered immediately inactive, negating the treatment effect. In a post hoc analysis of the data, subjects with metastatic disease who received STS had reduced EFS compared to those with metastatic disease who did not receive STS (42% vs. 61%) [49]. A clinical practice guideline was subsequently published, which recommended against the use of STS for prevention of cisplatin-induced ototoxicity in patients with metastatic cancer, which represents a large portion of HRNBL patients [50]. Although the guideline acknowledged the low quality of evidence given the susceptibility of post hoc analyses to bias, this has led to STS becoming standard treatment only in patients without metastatic disease, thus limiting the utility of STS in HRNBL patients. New preventative strategies are needed, as well as ongoing exploration of potential biomarkers that would identify those at greatest risk of hearing loss.

HRNBL survivors are at risk of infertility. This is most related to gonadal exposure to radiation and to chemotherapy with alkylating drugs. The Childhood Cancer Survivor Study (CCSS) is one of the largest long term follow-up cohorts and reported on over 5000 female survivors of childhood cancer including over 300 survivors of neuroblastoma and observed that survivors were less likely than their siblings to have become pregnant [51]. Similar findings were seen in the CCSS male cohort [52]. Because the majority of children with HNBL are prepubertal, fertility preservation strategies such as sperm banking and oocyte harvesting are not possible. Ovarian cryopreservation has recently become more common for fertility preservation in prepubertal girls and has now lead to about 200 live births and can be undertaken prior to treatment. Testicular tissue preservation, however, remains under investigation, with no live births known to have occurred using this strategy.

Children treated for HRNBL require comprehensive post-treatment follow-up and monitoring for toxicity, which is best carried in out in a multidisciplinary long-term survivorship follow up program. These programs are typically housed within pediatric oncology programs, though they may exist independently. In a recent large survey of COG-member institutions, 75% of programs reported they provide survivor care in a specialized late effects program, while 24% provide follow-up care in their regular pediatric oncology clinic. Despite the high proportion of institutions offering long-term follow-up, the majority of programs reported that fewer than 75% of eligible survivors were accessing this care [53]. Barriers that prevent survivors from engaging in the long-term follow-up are varied and include lack of knowledge, financial barriers, and psychosocial concerns [54]. Given the high prevalence of late effects in this population, life-long monitoring and follow-up is an important part of care for this group.

## 6. Health Care Disparities

Social determinants of health have been found to be associated with survival for patients being treated for HRNBL. A retrospective cohort study of children being treated with anti-GD2 antibody in COG trials found that children exposed to household poverty and both household and neighborhood poverty had inferior EFS and OS compared to those children who were not exposed to poverty [55]. A large population-based cohort study examining over 1200 children found that children in high-poverty counties and those with Medicaid had lower survival, and this discrepancy persisted over time when comparing children treated 1991–1998 with those treated 2011–2015 [56]. This may be related to access to resources, environmental factors, health literacy, or other socioeconomic factors. Importantly, data have shown that socioeconomic status significantly mediates disparities in childhood cancer survival among racial and ethnic groups in several cancers, including neuroblastoma [57]. Efforts to target socioeconomic factors and resources are paramount to ensuring all children have the best chance of cure.

## 7. Conclusions

Therapy for children with HRNBL is actively evolving, with research driving transitions to more targeted approaches. Standard therapy includes induction with cytotoxic chemotherapy and surgical resection, consolidation with ASCT and radiotherapy, and post-consolidation anti-GD2 immunotherapy. Incorporation of anti-GD2-based treatments into upfront therapy, during both the induction and post-consolidation setting, have shown encouraging preliminary results in clinical trials, though the impact on event-free and overall survival remain unknown. Current research efforts focus on defining the role of immunotherapy in treatment, reducing late effects, identifying novel treatment approaches, and improving the outcomes for patients with HRNBL.

## Figures and Tables

**Table 1:** 2017 International Neuroblastoma Response Criteria (INRC).

Overall Response	Response of Components Including Primary Tumor, Metastatic Disease, and Bone Marrow
Complete Response (CR)	CR in primary tumor, metastatic disease, and bone marrow
Partial Response (PR)	PR in at least one component and all others are better than stable disease
Minor Response (MR)	PR in at least one component, stable disease in at least one component, and no PD
Stable Disease (SD)	No component with better than SD. No PD
Progressive Disease (PD)	Any component with PD

**Table 2:** Clinical Trials with Novel Induction Regimens.

Standard COG Induction	5 Cycles	Intensive Chemotherapy
NCT03126916	6 cycles	5 cycles of intensive chemotherapy, 1 cycle of high dose 131I-MIBG
NCT04947501	4 cycles	Intensive chemotherapy
NCT03786783	5 cycles	2 cycles intensive chemotherapy, followed by 3 cycles of chemotherapy with dinutuximab + GMCSF
NCT01857934	6 cycles	Chemotherapy with hu14.18K322A + GMCSF + IL2

## Data Availability

No new data were created or analyzed in this study. Data sharing is not applicable to this article.
